# Patient Experience and Wound Healing Outcomes Using Different Palatal Protection Methods After Free Gingival Grafts: A Systematic Review

**DOI:** 10.3390/jfb15120360

**Published:** 2024-11-27

**Authors:** Tomasz Jankowski, Agnieszka Jankowska, Mirona Palczewska-Komsa, Maciej Jedliński, Wojciech Kazimierczak, Joanna Janiszewska-Olszowska

**Affiliations:** 1 Private Practice Dental Clinic Jankowscy, Czerwonego Krzyża 24, 68-200 Żary, Poland; agnieszkajankowska2301@gmail.com; 2 Department of Dental Prosthetics, Pomeranian Medical University in Szczecin, 71-111 Szczecin, Poland; mirona.palczewska.komsa@pum.edu.pl; 3 Department of Interdisciplinary Dentistry, Pomeranian Medical University in Szczecin, 71-111 Szczecin, Poland; maciej.jedlinski@pum.edu.pl (M.J.); joanna.janiszewska.olszowska@pum.edu.pl (J.J.-O.); 4 Kazimierczak Private Medical Practice, Dworcowa 13/u6a, 85-009 Bydgoszcz, Poland; w.kazimierczak@cm.umk.pl; 5 Department of Radiology and Diagnostic Imaging, Collegium Medicum, Nicolaus Copernicus University in Toruń, Jagiellońska 13-15, 85-067 Bydgoszcz, Poland

**Keywords:** free gingival graft, periodontology, platelet-rich fibrin, wound healing, pain perception, donor site

## Abstract

(1) Background: A free gingival graft (FGG) is a common technique used to reconstruct or enhance the area of keratinized mucosa, while a connective tissue graft (CTG) is utilized to boost soft tissue thickness, thereby promoting stability in interproximal marginal bone levels. Most reported complications following FGG procedure are associated with the donor site. In addition to a painful, open wound in the palate, the most frequent complications linked to FGG harvesting include excessive bleeding, postoperative bone exposure, and recurrent herpes lesions. Numerous methods for securing the donor site after a free gingival graft surgery have been documented in research publications. The main objective of this systematic review was to assess various techniques for protecting the palate after graft harvesting and their impact on patient experience, with a focus on pain management. The secondary objective was to evaluate these techniques in relation to donor site wound healing. (2) Methods: The search was performed across four databases: Medline (PubMed Central), Scopus, Web of Science, and Embase, in accordance with PRISMA guidelines and the recommendations set forth in the Cochrane Handbook for Systematic Reviews of Interventions. The initial search took place on 9 October 2023, followed by an update on 28 June 2024. The search utilized the following keywords: (“wound” OR “injury”) AND (“graft” OR “free gingival graft” OR “graft harvesting”) AND (“healing” OR “recovery”) AND “palate”. (3) Results: After conducting the follow-up search, a total of 958 papers were identified: 193 from PubMed, 314 from Scopus, 101 from Web of Science, and 350 from Embase. Ultimately, of the 49 papers that remained, 11 were excluded due to not fulfilling the inclusion criteria, leaving 38 full-text papers on free gingival grafts (FGG) for qualitative analysis. (4) Conclusions: Various methods for palatal protection after free gingival grafts (FGG) are described in the literature, stemming from biological, physical, or chemical sources. Most studies in this review examined platelet-rich fibrin and suggested that it provides no benefits for patients’ subjective experiences or wound healing outcomes. While photobiomodulation appears to support wound epithelialization, it does not influence pain perception. Alternatives such as propolis, hyaluronic acid, and medicinal plant extracts show potential for palatal protection; however, further research is needed to thoroughly evaluate their effectiveness.

## 1. Introduction

Dental implants, much like natural teeth, need the appropriate amount and quality of surrounding tissues, both soft and hard, to function properly. Given that implant therapy has become the standard approach for restoring edentulous areas and rehabilitating the masticatory system, the indications for gingival augmentation extend to both natural teeth and dental implants. Back in 1960, Clifford Ochsenbein noted that the attached gingiva is specifically designed for functional needs, whereas the alveolar mucosa, being thin and delicately attached tissue, is not adaptable for chewing function [1]. Lang and Löe [2] stated that gingival health can be maintained even with a very thin gingiva. Nevertheless, researchers suggested that in regions having less than 2 mm of keratinized mucosa, inflammation tends to persist despite effective oral hygiene. Therefore, they proposed that keratinized mucosa measuring a minimum of 2 mm (which is equivalent to 1 mm of attached gingival tissue in this situation) is sufficient to ensure periodontal health around the teeth. Additionally, it is worth noting that plaque management is expected to be more effective when there is more than 2 mm keratinized tissue zone present in the vicinity of the implant [3]. Sufficient tissue volume and width of keratinized gingiva appear to be essential determinants for peri-implant health [4].

**Figure 1:**
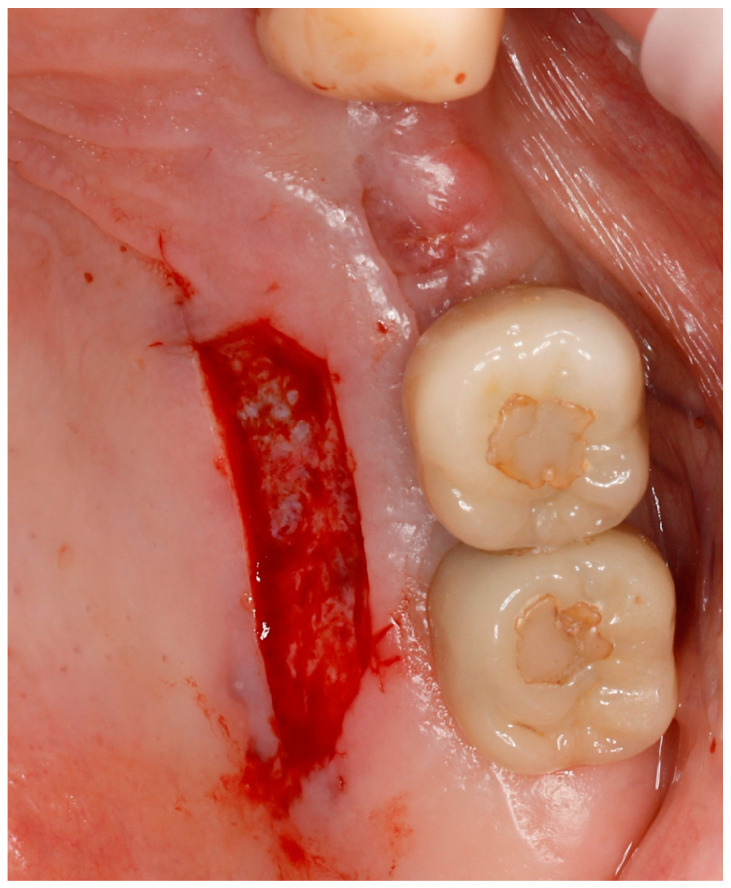
Photograph of a palatal wound created immediately after harvesting a free graft from the palate.

For almost sixty years, clinical periodontology has been defined by the use of autografts for two primary objectives: widening the keratinized mucosa and increasing soft tissue volume [5]. Autogenous graft-based approaches stand out as the most successful method for obtaining peri-implant soft tissue improvement [4]. A free gingival graft (FGG) is a common technique for rebuilding or expanding the keratinized mucosa area, while a connective tissue graft (CTG) serves to augment soft tissue volume, consequently promoting stability of interproximal marginal bone levels [3]. The majority of documented complications following FGG or CTG procedures are connected with the donor site [6] (Figure 1). Apart from a painful, open wound of the palate, the most common complications related to harvesting of FGG are as follows: excessive bleeding, postoperative bone exposure, and recurrent herpes lesions [7].

Various methods of securing the donor site following FGG procedure have been described in the literature. The absorbable gelatin sponge appears to serve as the standard method for protecting the palate following free gingival graft (FGG) procedures, as evidenced by its consistent use in the control groups of several investigations [8,9,10]. The addition of cyanoacrylate and hyaluronic acid to a gelatin sponge has shown significant benefits in protecting the palatal donor site [10]. Furthermore, the use of platelet-rich fibrin (PRF) as a protective agent in palatal donor sites during free gingival graft (FGG) procedures has been associated with reduced postoperative pain and accelerated epithelialization of the wound [11]. Moreover, laser photobiomodulation following FGG appears to promote epithelialization at the donor site, although it has limited effect on reducing postoperative pain [12]. These examples represent only a few of the methods for protecting the palate following graft harvesting and their respective impacts on the healing process.

While systematic reviews on specific methods of palate protection are present in the literature, a comprehensive review synthesizing all available techniques for protecting the palatal donor site has not yet been conducted. Such a review would be valuable in summarizing the effects of these methods on patient experience and wound healing outcomes, while also addressing the complications commonly associated with donor site management. The main objective of this systematic review was to assess various techniques for protecting the palate after graft harvesting and their impact on patient experience, with a focus on pain management. The secondary objective was to evaluate these techniques in relation to donor site wound healing.

## 2. Materials and Methods

The systematic review was registered in the INPLASY database (https://inplasy.com/) on 25 November 2024, with the following registration DOI: https://doi.org/10.37766/inplasy2024.11.0107.

### 2.1. Eligibility Criteria

#### 2.1.1. Inclusion Criteria

The PICO components were applied in this systematic review as formulating the right questions is essential for such reviews [13]. The questions were as follows:

1. Patients (P): individuals who underwent harvesting of FGG or deepithelialized CTG;
2. Intervention (I): preservation of the palatal graft site using methods other than gauze compression, suturing, or gelatin sponge;
3. Comparison (C): preservation of the palatal graft site using standard methods like gauze compression or gelatin sponge;
4. Outcome (O): wound healing, pain assessment, postoperative discomfort, sensory disturbance, color matching, and secondary bleeding.

The inclusion criteria were defined as:

1. Studies classified as randomized clinical trials;
2. Publications written in the English language;
3. Studies focusing on patients aged 18 years or older;
4. Research involving patients who underwent the removal of a free graft from the palate, with the harvested area protected by various methods in the test group and an alternative healing technique in the control group.

#### 2.1.2. Exclusion Criteria

The exclusion criteria were defined as:

1. Studies without a control group;
2. Research involving donor sites outside the palatal area;
3. Research examining grafting techniques other than free gingival grafts (FGG) and de-epithelialized connective tissue grafts (CTG);
4. Studies not written in English;
5. Book sections, expert opinions, guidelines, letters, conference materials, animal research, literature reviews, case reports, case series, abstracts, debates, or editorials.

### 2.2. Search Strategy

The search was carried out across four databases—Medline (PubMed Central), Scopus, Web of Science, and Embase—adhering to the PRISMA (Preferred Reporting Items for Systematic Reviews and Meta-Analyses) [14] and the instructions outlined in the Cochrane Handbook for Systematic Reviews of Interventions [15]. The PRISMA 2020 manuscript checklist is provided in the Supplementary Materials. The initial search was carried out on 9 October 2023, with a follow-up on 28 June 2024 without restrictions on publication date. Both searches utilizing a mix of MeSH terms, free-text keywords, and subject headings in both instances. The search strategy employed the following keywords: (“wound” OR “injury”) AND (“graft” OR “free gingival graft” OR “graft harvesting”) AND (“healing” OR “recovery”) AND “palate”.

### 2.3. Data Extraction

The titles and abstracts obtained from the search were individually assessed and chosen for further examination by two reviewers (T.J. and M.J.). The full text of each selected primary article was evaluated to verify its adherence to the eligibility criteria. Discrepancies were settled by consulting the team coordinator (J.J.O.). Data including authorship, publication year, ethnic group, type of palate protection, and methods used to evaluate the effectiveness of the palate protection technique were independently extracted by the two authors (T.J. and M.J.) and validated by the team coordinator (J.J.O.).

### 2.4. Risk of Bias

The methodological risk assessment of bias for each study was performed by two independent authors (M.P.K. and T.J.), and, in the case of disagreement, it was resolved by a third author (J.J.O). For randomized clinical trials (*n* = 38), the qualitative analysis of the studies was performed based on the risk of bias assessment using the Cochrane risk of bias tool for randomized clinical trials (RoB 2): ‘Bias Risk Assessment of Randomised Controlled Studies’—*Cochrane Handbook 6.0* [16]. The following domains were considered:

1. Randomization process;
2. Deviations from intended interventions;
3. Missing outcome data;
4. Measurement of the outcome;
5. Selection of the reported results.

The blinding of operators was not considered since it was impossible to perform in these types of interventions. Each study included was assessed as having a ‘high’ risk of bias for domains with a negative response (red), a ‘low’ risk of bias for those with a positive response (green), and an ‘unclear’ risk of bias (yellow) when the response was ambiguous. Overall quality was based on the scores in individual domains; the overall quality was of low bias risk. When at least one domain had an uncertain level of risk, the overall quality was deemed to have an unclear risk of bias. Also, the assessment of at least one domain as high risk or three or more domains as having unclear risk resulted in a quality of high risk of bias.

## 3. Results

### 3.1. Search Results

**Figure 2:**
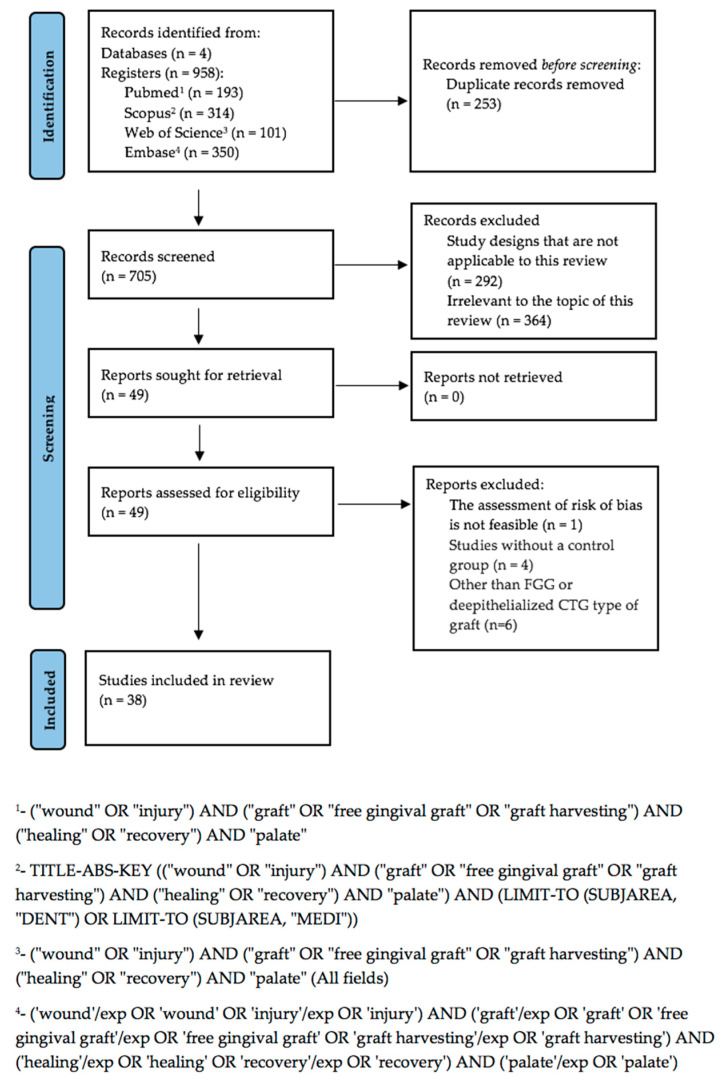
Prisma 2020 flow diagram.

After performing a follow-up search, 958 papers were identified: 193 from PubMed, 314 from Scopus, 101 from Web of Science, and 350 from Embase. Using Mendeley software (v2.119.0), 253 duplicates were eliminated, leaving 705 abstracts for the first screening phase. At this stage, 292 articles were excluded as they did not align with the study type outlined in the eligibility criteria. From the 413 remaining titles, 364 were discarded for being irrelevant to the topic of this review. Ultimately, out of the 49 remaining papers, 11 were excluded for failing to meet the inclusion criteria, resulting in 38 full-text papers on free gingival grafts (FGG) being retained for qualitative analysis. The process is illustrated in Figure 2 (PRISMA 2020 Flow Diagram).

Data regarding various methods of palatal protection following the harvesting of free gingival grafts (FGGs) were complied with postoperative parameters and extracted. Table 1 provides a summary of the characteristics of each study included.

The most frequently studied method of palatal protection is platelet-rich fibrin (PRF) [21,22,26,30,31,32,37,43,50,53], followed by photobiomodulation (PBM) [20,23,28,38,45]. In the majority of studies [19,21,23,24,25,26,28,29,30,31,33,34,35,36,38,39,40,41,42,43,45,46,47,49,50,51,54], pain is measured using the visual analog scale (VAS) developed by Huskisson [55]. The VAS is also utilized to assess color match [19,21,23,25,29,36,46], the alteration of sensitivity [22], postoperative discomfort [22,24,28,29,45,49,50], changes in patients’ feeding habits [22,28,29,47], or burning sensations [23,25,28,29,47,49]. Epithelialization is the most frequently evaluated healing parameter, with authors assessing it using 3% hydrogen peroxide (bubbling test) [19,21,22,26,27,28,31,34,40,45,46,47,49] or through standard clinical photographs [25,39,42,45]. A dedicated summary of the research findings for platelet-rich fibrin (PRF) and photobiomodulation (PBM) methods is provided in Table 2 and Table 3. The authors of this review (T.J. and J.J.O.) categorized the methods of palatal protection into three groups: biological, physical, and chemical. This classification is detailed in Table 4.

### 3.2. Risk of Bias

**Figure 3:**
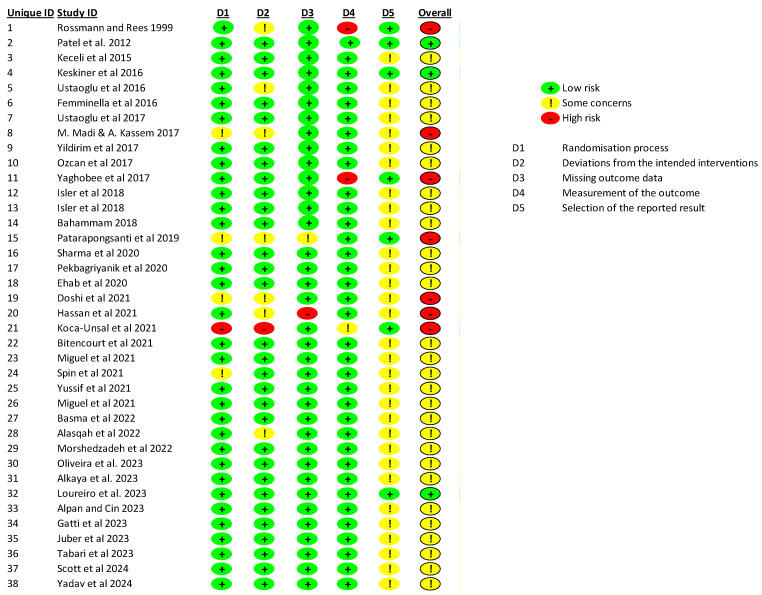
The qualitative analysis for randomized clinical trials [17,18,19,20,21,22,23,24,25,26,27,28,29,30,31,32,33,34,35,36,37,38,39,40,41,42,43,44,45,46,47,48,49,50,51,52,53,54].

The database search yielded a total of 958 records. However, it is important to note that not all databases supported the application of the full set of inclusion and exclusion criteria. As a result, during the next stage of selection, studies that did not meet the established criteria were manually excluded, and duplicates were removed. After a thorough review based on the inclusion criteria, 38 studies were deemed eligible for analysis (Figure 3).

In the randomization process, 4 publications were labelled as having some concerns, and one publication was high risk, while the remaining publications were low risk. In domain no. 2 (deviations from the intended interventions), five publications were labelled as having some concerns, one publication was high risk, and the remaining publications were low risk. In the domain concerning missing outcome data, one publication had some concerns and one publication had high risk, while the remaining publications had low risk. In domain 4 (measurement of the outcome), one publication had some concerns, two had high risk, and the remaining publications had low risk. In terms of selecting the reported results, the majority, with as many as 31 publications, raised concerns primarily related to the use of subjective assessments of parameters such as pain, post-procedure discomfort, and the amount of painkillers consumed. Domain 5 significantly influenced the overall risk of bias score, in which seven publications had high risk status, only three publications had low risk status, and the remaining publications had some concerns.

## 4. Discussion

### 4.1. Quantification and Evaluation of Postoperative Pain

Pain is the most frequent complication observed after palatal graft harvesting [56]. Therefore, from the patient’s perspective, the most important aspect is to assess postoperative pain using various methods of palate protection. The degree of pain experienced at the donor site does not significantly impact the clinical outcomes of the treatment; nevertheless, it plays a critical role in determining the patient’s willingness to consent to similar procedures in the future. The main challenge encountered by the authors of this review is that factors such as pain perception, burning sensations, post-surgery discomfort, and changes in patients’ eating habits are all inherently subjective. One issue with these factors is their subjective nature, and another is that they are assessed using various methods. In the studies included, a visual analog scale (VAS) is frequently used to evaluate these subjective parameters [19,21,22,23,24,25,26,28,29,30,31,33,34,35,36,38,39,40,41,42,43,45,46,47,49,50,51,54]. For example, when assessing pain levels, 0 represents no pain, while 10 indicates the highest degree of pain.

#### 4.1.1. Platelet-Rich Fibrin (PRF)

In 4 out of 10 publications [26,30,31,43] comparing PRF with other standard methods of palate protection, PRF demonstrated an advantage in terms of pain perception. Following this, only Basma et al. [43] noted that patients who had PRF protection on the palate utilized fewer analgesics. None of the articles examining the effect of photobiomodulation on the wound after graft removal showed an improvement in pain perception [23,28,38,45], though two studies reported a reduction in postoperative discomfort [28,38]. Rossmann and Rees [17] compared oxidized regenerated cellulose, absorbable gelatin sponge, and sterile gauze compression, finding no differences in pain assessment across the treatment groups.

#### 4.1.2. Ozone

Ozone is extensively applied in medical practice post-surgery, as it enhances microcirculation, stimulates the proliferation of immunocompetent cells, and facilitates the increased secretion of growth factors [57]. Isler et al. [28] compared ozone therapy and photobiomodulation with a control group (spontaneous healing) and found that, although the control group showed higher VAS scores for postoperative pain, there was no statistically significant difference between the groups. Additionally, no differences were observed in terms of analgesic consumption, dietary habits, or burning sensations. However, patient discomfort was significantly greater in the control group.

#### 4.1.3. Chitosan Gel and Propolis

Chitosan gel and propolis, both derived from animal sources, offer a wide range of applications in the medical field. Chitosan demonstrates antimicrobial activity against fungi and bacteria due to its cationic nature [58]. Yadav et al. [54] found no statistically significant differences in pain levels, analgesic consumption, dietary changes, or sensory alterations between chitosan-based dressings and the control group, which used wet gauze. In a separate study, Madi and Kassem [24] evaluated four groups: simvastatin suspension, simvastatin combined with chitosan gel, chitosan gel alone, and petroleum gel. While chitosan gel alone did not provide any notable benefit in reducing pain intensity or discomfort in palatal wounds, the combination of simvastatin with chitosan gel significantly lowered VAS scores compared to the other three groups. Propolis, on the other hand, is a resinous material created by bees, recognized for its antioxidant, anti-inflammatory and antimicrobial properties, which is effective against bacteria and fungi, much like chitosan [59]. Tabari et al. [52] demonstrated that a 5% propolis oral ointment significantly lowered pain scores compared to a placebo, though it had no significant impact on burning sensation. Similarly, other studies have reported reduced pain in patients with palatal wounds treated with propolis [44].

##### 4.1.4. Alternative Approaches for Evaluating Subjective Outcomes

This systematic review also incorporated studies utilizing alternative methods to the VAS for assessing subjective sensory outcomes. Scott et al. [53] evaluated pain and analgesic intake using the 21-point numerical rating scale (NMRS-21) in patients from both the PRF and hemostatic agent groups, with no significant differences observed between them. Another assessment tool is the Oral Health Impact Profile-14 (OHIP-14), which consists of 14 questions addressing oral and general health concerns experienced over the preceding 12 months, spanning seven dimensions: functional limitation, pain, psychological discomfort, physical and psychological disability, social disability, and disadvantage [51]. Juber et al. [51] reported that participants treated with highly ozonated sunflower oil exhibited higher overall OHIP-14 scores compared to the control group. Different researchers have also utilized OHIP to evaluate the efficacy of palate protectors [35,39].

### 4.2. Wound Healing Assessment

Techniques for evaluating wound healing after graft removal seem more reliable, as their assessments are independent of the patient and performed by the researcher. However, the variety of healing assessment methods used in this review complicates the objective comparison of the included studies. The “bubble test” is one of the most frequently employed methods to assess wound healing, utilizing hydrogen peroxide. The principle behind this test is that when the epithelium is not intact, the hydrogen peroxide penetrates the connective tissue, where the enzyme catalase breaks it down into water and oxygen [21]. This reaction is clinically indicated by the formation of bubbles on the wound surface [21]. In the included studies, the authors have defined the healing parameter using the bubble test in various ways, such as epithelization (EP) [17,19,27,31,40], complete wound epithelization (CWE) [21,22,35,46], complete epithelization (CE) [26], complete reepithelization [34,47,49], or remaining wound area (RWA) [28]. Regardless of the nomenclature, all of these parameters ultimately evaluate the extent of the wound surface covered by epithelium within a specified period following the procedure. Palatal wound healing can also be evaluated through the analysis of standardized photographs, where the wound area is measured in square millimeters (mm^2^) using specialized software or visual observation [25,26,35,39,42,45].

Studies comparing PRF (platelet-rich fibrin) with control groups demonstrated a healing advantage of PRF in palatal wound healing in five studies [21,22,26,31,37], while two studies [32,53] reported a disadvantage. In the case of applying photobiomodulation, the authors agree that it provides benefits for wound healing [23,28,38,45]. Keceli et al. [19] assessed epithelialization (EP) weekly for the first month using bubble formation after applying 3% hydrogen peroxide, ranking EP as total, partial, or none, and found that medicinal plant extract (MPE) promoted better healing compared to the wet gauze group. Rossmann and Rees [17] demonstrated that oxidized regenerated cellulose (ORS) promotes superior palatal wound healing compared to an absorbable gelatin sponge. In contrast to the conclusions of Scott et al. [53], Patarapongsanti et al. [31] demonstrated a significant advantage of PRF over ORC in promoting wound healing. Pekbagriyanik et al. [33] reported that by the second week, the number of patients achieving complete epithelialization was higher in the non-thermal atmospheric pressure plasma (NAPP) group compared to the control group. Yadav et al. [54] determined that chitosan-based dressings exhibited a significantly higher rate of complete epithelialization (CE) at both the second and third weeks compared to the control group using wet gauze. Madi and Kassem’s study [24], which evaluated four groups (simvastatin suspension, simvastatin combined with chitosan gel, chitosan gel alone, and petroleum gel), demonstrated a significant advantage of the simvastatin combined with chitosan gel at 3 and 7 days; however, no significant differences were noted at the 14-day postoperative evaluation. Yildirim et al. [25] reported that hyaluronic acid (HA) gels accelerate epithelialization at the palatal donor wound site. In the investigation by Oliveira et al. [46], three groups were compared: a sutures group, a placebo group, and a group receiving a gel containing green tea extract and hyaluronic acid; nonetheless, no significant differences were found among the groups concerning complete wound epithelialization.

Alpan et al. [49], in their study, employed the Landry Wound Healing Index (WHI) along with the bubbling test to assess wound healing from day 3 to day 28 postoperatively. This index, scored from 1 (very poor) to 5 (excellent), evaluates wound healing based on tissue color, palatal response, granulation tissue presence, epithelialization at incision edges, and the degree of suppuration. The results of their investigation indicated that hyaluronic acid treatment produced higher WHI values compared to treatment with hypochlorous acid and flurbiprofen from the 7th to the 21st day. Complete epithelialization was observed on day 21 in the hyaluronic acid group, while it occurred on day 28 in the other groups.

## 5. Conclusions

(1) The literature documents numerous techniques for palatal protection following free gingival grafts (FGG), utilizing materials of biological, physical, or chemical origin.
(2) This systematic review included 10 publications on PRF. Five of these showed a healing benefit from PRF, while four indicated an advantage in pain perception.
(3) Photobiomodulation evidently enhances wound epithelialization, yet has no effect on pain perception.
(4) Propolis, hyaluronic acid, or medicinal plant extracts are promising alternatives for palate protection; however, there is insufficient research to fully assess their effectiveness.
(5) The application of a gelatin sponge stabilized with sutures is widely regarded as a standard method for protecting the palatal donor site following periodontal grafting procedures. However, the results of the aforementioned studies underscore the need for further investigation into palatal wound protection techniques to definitively determine the clinical advantages and patient outcomes associated with specific methods.

## Figures and Tables

**Table 1:** Characteristics of included studies.

Author, Year of Publication	Type of Study	Objectives	Number of Subjects	Evaluation	Age Range (Years)	Results
Rossmann and Rees, 1999 [17]	Single center, prospective, randomized, controlled, open-label evaluation	To assess the effectiveness of three techniques for achieving hemostasis on the palate following donor-tissue-harvesting for autogenous soft tissue grafts.	26 American patients: - Oxidized regenerated cellulose—ORC (*n* = 9); - Absorbable gelatin sponge (*n* = 9); - Sterile gauze with external pressure served as the control method (*n* = 8).	The percentage of epithelial coverage over the wound at each visit, along with the status of wound healing (normal or abnormal). Pain assessments were conducted on day 7 and at the patient’s final visit (0–10 scale). The amount of pain medication.	40–46 (mean age)	The median time to achieve hemostasis was notably shorter with the application of a hemostatic agent, compared to the control groups in both sets. Pain evaluations showed no significant differences between the treatment groups. By day 21, only the ORC group exhibited complete healing, with all sites classified as normal or rapidly healing, while in the absorbable gelatin sponge group, 40% of the sites were categorized as slow healing. “Bleeding was primarily observed during the first 7 days in 40% of both the ORC and control groups, whereas no adverse events were reported in the absorbable gelatin sponge group.
Patel et al., 2012 [18]	Randomized, placebo-controlled clinical trial with triple blinding.	To evaluate the therapeutic impact of topical ozonated oil on the early healing of FGG surgical sites.	20 Indian subjects: - Intervention ozone group (*n* = 10); - Control group (*n* = 10).	The cytological analysis included the measurement of keratinization and superficial cell indices at baseline.	22–35	Significant improvement in epithelial healing was observed in the ozone group at the 7th, 14th, and 21st days, as well as at 2, 3, and 8 months postoperatively, compared to the control group.
Keceli et al., 2015 [19]	Single-centered, randomized, prospective and controlled study	To assess the effectiveness of medicinal plant extract (MPE) in achieving hemostasis at the donor site.	33 Turkish patients: - Control group (*n* = 16)—WG (wet gauze) group; - Test group (*n* = 17)—MPE + WG group.	Primary bleeding time. Questionnaires: - Number of medications; - VAS pain scores; - Presence of secondary bleeding (BLE). Epithelization (EP) using 3% hydrogen peroxide (total/partial/none). Color match (CM)—VAS scores.	22–40	In the test group, the primary BLE duration was shorter, and fewer individuals experienced secondary BLE over the three-day period. Pain scores were higher in the WG group during the six-day period. EP occurred more quickly, and CM showed slight improvement in the MPE + WG group.
Keskiner et al., 2016 [20]	Double-blind, parallel, randomized controlled clinical trial	To assess the effect of photobiomodulation (PBM) on healing in the donor palatal area after FGG harvesting by analyzing changes in transforming growth factor (TGF)-ß1, platelet-derived growth factor (PDGF)-BB, and interleukin (IL)-8 levels in palatal wound fluid (PWF).	30 Turkish patients: - Laser group (*n* = 15); - Sham group (*n* = 15).	Palatal wound fluid (PWF) samples were obtained on days 7 and 12 after surgery, utilizing periopaper strips. PWF sample volume was measured using a Periotron 8000. PWF levels of TGF-ß1, PDGF-BB, and IL-8 were measured using enzyme-linked immunosorbent assays (ELISA).	20–31	On day 12, PWF levels of TGF-ß1, PDGF-BB, and IL-8 were significantly lower than on day 7 for both groups. On day 7, the PWF levels of TGF-ß1, PDGF-BB, and IL-8 in the laser group were significantly higher compared to the sham group. On day 12, the PWF TGF-ß1 levels were also significantly elevated in the laser group.
Ustaoglu et al., 2016 [21]	Single-center, randomized, prospective controlled study	To assess the clinical effects of titanium-prepared platelet-rich fibrin (T-PRF) on the healing of human palatal mucosal wounds (PMWH) and to examine its impact on time-related changes in palatal soft tissue thickness (PSTT) associated with the process of histoconduction	34 Turkish patients: - T-PRF group (*n* = 16); - Control group (*n* = 18).	Color match (VAS score). CWE—H_2_O_2_ bubbling test. Pain levels (VAS score), the number of analgesics taken, and bleeding status (yes/no) were documented during the first 7 days. PSTT was assessed at baseline, and again at 1 and 6 months.	No data	The color match scores of the test group were significantly higher than those of the control group at both 7 and 14 days. CWE occurred more frequently in the test group than in the control group on day 14. The prevalence of postoperative bleeding was lower in the test group during the first 2 days. A time-dependent reduction in PSTT was observed at 1 and 6 months in the control group compared to baseline, whereas no significant change was noted in the test group.
Femminella et al., 2016 [22]	Randomized controlled clinical trial (RCT) with a prospective, parallel design	To evaluate and compare the effects of PRF and gelatin sponge on the healing process of palatal donor sites and the associated patient morbidity.	40 Italian patients: - Test group (*n* = 20)—the palatal wound was covered with a four-layer PRF dressing; - Control group (*n* = 20)—the wound was medicated by an absorbable gelatin sponge.	Primary outcome—the time taken to achieve complete re-epithelialization of the palatal wound (CWE), as assessed using the peroxide test. Secondary outcomes using VAS: - “The alteration of sensitivity (AS)”; - “The postoperative discomfort (D)”; - “Changes in patients’ feeding habits (CFH)”; - “The consumption of analgesics”; - “The existence of delayed bleeding (DWB)”.	18–47	The test group exhibited a significantly quicker CWE, with 35% of test patients achieving CWE by the end of week 2 (compared to 10% in the control group). By the end of week 3, all palatal wounds in the test group had fully epithelialized, in contrast to just 25% of wounds in the control group. Test patients experienced significantly less discomfort and CFH, and required a considerably lower dose of analgesics.
Ustaoglu et al., 2017 [23]	Prospective, randomized, double-blind, single-center, controlled clinical study	To evaluate the impact of low-level laser therapy (LLLT) on the healing of wounds at FGG donor sites.	35 Turkish patients: - Control group (*n* = 18)— sham LLLT; - Test group (*n* = 17)—LLLT.	Intra-surgical measurements of soft TT—endodontic reamer. Diary of pain and burning sensations, color match (VAS). Bleeding (+/−). Palatal tissue consistency. CWE—peroxide bubbling test. Landry Wound-Healing Index (WHI).	25–45	On the 14th day, the prevalence of CWE was greater in the LLLT group compared to the control groups. Bleeding was less frequent in the test group than in the control group during the first 2 days. The test group showed higher WHI scores than the control group at all follow-up visits. Color match scores were higher in the test group compared to the control group at the first three visits.
M. Madi & A. Kassem, 2017 [24]	No data	To investigate the impact of applying simvastatin/chitosan gel (10 mg/mL) topically to the palatal donor site following the FGG procedure.	40 Egyptian patients: - Group I (*n* = 10)—simvastatin suspension (S); - Group II (*n* = 10)—simvastatin/chitosan gel (SC); - Group III (*n* = 10)—chitosan gel (C); - Group IV (*n* = 10)—petroleum gel.	Wound healing—clinical observation and scores were determined (“4-necrotic tissue, 3-slough, 2-granulation tissue, 1-epithelial tissue, 0-closed”). Intensity of pain and discomfort—VAS. Analgesic consumption.	25–40	Group II showed a statistically significant reduction in wound healing scores after 3 and 7 days when compared to the other groups”. A significant decrease in the VAS score was also noted for group II compared to the other groups on days 1, 3, 5, and 7.
Yildirim et al., 2017 [25]	Randomized, controlled, prospective clinical superiority trial with a parallel-group design and examiner-blinding	To examine the effects of two concentrations of topical hyaluronic acid on postoperative discomfort and healing of palatal donor sites following FGG surgery.	36 Turkish patients: - Test-1 group (*n* = 12)—0.2% hyaluronic-acid gel; - Test-2 group (*n* = 12)—0.8% hyaluronic-acid gel; - Control group (*n* = 12).	Pain and burning sensations were assessed using the visual analog scale (VAS). Complete epithelization (CE)—means of clinical photographs. Color match (CM)—VAS.	21–62	The test groups reported less pain than the control group on days 3 and 7. On day 3, the mean VAS score for burning sensation was higher in the control group compared to the test groups. Complete epithelialization (CE) was achieved by day 21 in both test groups, while it occurred on day 42 in the control group. The test groups had higher color match scores than the control group on both days 21 and 42.
Ozcan et al., 2017 [26]	Single-center, prospective randomized, controlled evaluation	To assess the impact of PRF on the healing of palatal wounds following FGG harvesting.	125 Turkish patients: - PRF group (*n* = 42)—“PRF with butyl-cyanoacrylate (BC) adhesive”; - BC group (*n* = 42)—“BC adhesive alone”; - WG group (*n* = 41)—“sterile wet gauze compression”.	Delayed bleeding (DB)—7-day evaluation. CE—use of color photographs and H_2_O_2_ to observe bubbling (once a week during the first month). Sensibility disorders were evaluated using a periodontal probe—6-week follow-up. Feeding habits (FH)—4-week follow-up. Pain perception (VAS)—28-day follow-up.	21–48	All parameters showed statistically significant differences, with the PRF group showing better outcomes.
Yaghobee et al., 2017 [27]	Triple-blind, randomized, placebo-controlled clinical trial with a split-mouth design	To assess the impact of topical erythropoietin (EPO) on the healing process of the donor site.	12 Iranian patients with insufficient attached gingiva at least at 2 sites in the mandible: - Test group (*n* = 12)—EPO group; - Control group (*n* = 12)—vehicle gel group.	Epithelialization—3% H_2_O_2_. Healing rate—direct observation and observation of photographs.	30–53	The EPO group demonstrated significantly better keratinization only on day 21. Direct examination of clinical healing showed significantly better healing in the test group on day 28. Inflammation was lower in the test group compared to the control group on the same day.
Isler et al., 2018 [28]	Prospective, randomized, controlled clinical trial with a parallel design and examiner blinding	To assess the effects of photobiomodulation and topical ozone therapy on the reepithelialization process of palatal wounds following FGG surgeries.	36 Turkish patients: - Laser group (*n* = 12)—low-level laser therapy (LLLT); - Ozone group (*n* = 12)—ozone therapy; - Control group (*n* = 12)—palate was left for spontaneous healing.	Primary outcome—remaining palatal wound area (mm^2^) by applying 3% H_2_O_2_. Secondary outcome: Using VAS scale questionnaire: - “Postoperative pain”; - “Patient discomfort”; - “Changes in patient dietary habits”; - “Presence of burning sensation”; Amount of systemic analgesic consumed.	27–51	On day 14, digital image analysis showed significantly smaller wounds in the ozone group compared to the control group. However, intergroup comparison using the H2O2 method did not show any significant differences. On day 7, VAS scores were significantly higher in the control group compared to both the laser and ozone groups.
Isler et al., 2018 [29]	Randomized, prospective, double-blind, placebo-controlled study with a parallel design	To assess whether the use of an oral flurbiprofen spray reduced patient pain perception and improved patient morbidity following palatal graft harvesting.	48 Turkish patients: - Test group (*n* = 24)—flurbiprofen (SCTG/FGG); - Control group (*n* = 24)—placebo (SCTG/FGG).	Intraoperative parameters: - “Immediate bleeding (IB)—yes/no”; - “Graft thickness (GT)”; - “Graft width (GW)”; - “Graft height (GH)”. Postoperative parameters—VAS: - “Postoperative pain”; - “Patients discomfort”; - “Changes in dietary habits”, - “Burning sensation”; - “Color match”.	30–52	At 21 days postoperatively, the prevalence of complete epithelialization was significantly higher in the placebo FGG group compared to the flurbiprofen-FGG group. In the flurbiprofen-FGG group, significant improvements were noted in postoperative pain, patient discomfort, and burning sensation by 14 days postoperatively.
M. A. Bahammam, 2018 [30]	Clinical trial designed as a prospective, randomized study	To assess whether applying a platelet-rich fibrin (PRF) palatal bandage after harvesting a free gingival graft (FGG) could enhance donor site healing, reduce pain levels, and minimize patient discomfort.	24 Saudi patients: - Test group (*n* = 12)—PRF; - Control group (*n* = 12).	Pain assessment for 7 days: - VAS; - NRS-101—101-point numerical rating scale; - VRS-4—four-point verbal rating scale. Analysis of clinical results (8-week evaluation): - Color changes; - Contour changes; - Texture changes.	18–40	Patients in the PRF group reported significantly lower pain scores and experienced a quicker return to baseline pain levels compared to those in the control group. PRF facilitated wound healing after FGG.
Patarapong-santi et al., 2019 [31]	Clinical trial	To compare patient morbidity and healing outcomes between platelet-rich fibrin (PRF) and oxidized regenerated cellulose (ORC) on palatal donor sites following free gingival graft (FGG) harvesting.	18 Thai patients with the need for bilateral FGG: - Test group (*n* = 18)—PRF; - Control group (*n* = 18)—ORC.	H_2_O_2_ test—integrity of epithelialization. Postoperative pain—VAS score. Percentage of wound healing—using digital camera and software.	45–78	1 week—similar wound size reduction in both groups. By the two-week mark, most of the test group (88.89%) had achieved complete epithelialization (CE), while 66.67% of the control group showed CE. On day 1, pain was reported more frequently in the control group (27.7%) than in the test group (11.1%). No participants reported any pain or discomfort at the test sites by day 3.
Sharma et al., 2020 [32]	Randomized parallel-designed human clinical trial	To compare the efficacy of a commercially available collagen dressing (CollaCote^®^) with autologous PRF membrane as a palatal protection to promote wound healing at the donor site following a free gingival graft (FGG).	20 Indian patients: - Group 1—collagen membrane (*n* = 10); - Group 2—PRF membrane (*n* = 10).	Primary outcomes: - Depth and size of the wound; - Epithelialization Tests: conducted using the hydrogen peroxide test and toluidine blue test over a 31-day period. Secondary outcomes: - Immediate or delayed bleeding during 7 days; - Pain/discomfort postoperatively—evaluated over a 30-day period.	18–52	Intragroup comparisons revealed a significant improvement in wound healing parameters for both groups. No significant differences were observed between the groups in terms of depth, hemorrhage, pain, epithelialization, and size, although the PRF group showed slightly better healing initially.
Pekbagriyanik et al., 2020 [33]	Randomized, prospective, controlled clinical study with parallel arms	To assess the impact of non-thermal atmospheric pressure plasma (NAPP) on wound healing, epithelialization, local pain, bleeding, and changes in sensation at the palatal donor site.	36 Turkish participants: - Test group (*n* = 18)—FGG + NAPP; - Control group—FGG alone.	Questionnaire: - Pain—VAS; - Total number of drugs taken; - Presence of bleeding (yes/no). The following parameters were recorded: - “Complete reepithelization”; - “Alteration of sensation around the palatal wound”; - “Color match (CM)”.	30–51	By the second week, a higher number of patients in the NAPP group had achieved complete epithelialization compared to the control group. The color match at the donor site was more favorable in the NAPP group than in the control group during the first five follow-up assessments. No significant differences were observed between the two groups in terms of bleeding, pain levels, medication use, or changes in sensation.
Ehab et al., 2020 [34]	Prospective, randomized, and controlled clinical trial utilizing a parallel design	To compare the effects of Alvogyl and absorbable gelatin sponge as palatal wound dressings on postoperative pain, analgesic use, post-surgical bleeding, and wound reepithelialization.	36 Egyptian patients: - Intervention group (*n* = 18)—Alvogyl; - Control group (*n* = 18)—absorbable gelatin sponge.	Primary outcome: - patient-reported daily pain scores on the VAS scale over a 2-week period. Secondary outcomes: - post-surgical bleeding; - complete re-epithelization during the 5-week follow-up period (3% H_2_O_2_); - the number of analgesic tablets taken during the first week.	22–41	The control group reported significantly higher VAS pain scores up to 12 days post-surgery, accompanied by increased analgesic consumption. However, a multivariate regression analysis, accounting for factors such as age, gender, graft dimensions, tissue thickness, analgesic use, and dressing type, revealed no statistically significant impact of any factor, including dressing type, on VAS pain scores. At 4 weeks, 22.2% of patients in the test group achieved complete re-epithelialization, compared to 11.1% in the control group. Both groups reached complete re-epithelialization by 5 weeks.
Doshi et al., 2021 [35]	Prospective split-mouth clinical study	To evaluate clinical, histological, and patient outcomes after the topical application of phenytoin (PHT) to experimental palatal wounds.	20 American subjects: - PHT side (*n* = 20)—10% phenytoin USP; - Control side (*n* = 20)—received carrier alone.	Clinical assessments: - “Healing Score Index (HSI)”—assessed over a 21-day period. - “Wound Size and CWE”—visual assessment and the peroxide test carried out on the 14th and 21st days after surgery. Patient-centered outcomes (evaluated over a 21-day period): - “Oral Health Impact Profile-14 (OHIP-14)”; - Pain experience, analgesic use and postoperative complications—“VAS” and “Functional Pain Scale (FPS)”. Histomorphometric assessments (Biopsies collected from anterior wounds on day 1 or day 5).	23–31	On day 1, 30% of participants reported more pain on the control side compared to the PHT-treated side. The PHT-treated sides were more likely to show no swelling and no pain upon palpation. PHT treatment significantly influenced the appearance of granulation tissue in a time-dependent manner. Histological analysis revealed no significant differences between the control and PHT-treated sides at any time point.
Hassan et al., 2021 [36]	Randomized clinical trial	To evaluate and compare the effects of MEBO (Moist Exposed Burn Ointment) and 0.2% hyaluronic acid gel (Gengigel^®^) on decreasing postoperative pain and promoting wound healing at the palatal donor site after free gingival graft (FGG) harvesting.	39 Egyptian patients: - “Group I—MEBO” (*n* = 13); - “Group II—0.2% HA” (*n* = 13); - “Group III—control” (*n* = 13).	Primary outcome—for 7 days: - Postoperative pain—VAS and mean consumption of analgesics. Secondary outcome: - Wound size—measurement using periodontal probe over a 21-day period; - Color match—VAS (42-day assessment).	24–49	Postoperative pain: MEBO demonstrated a significantly lower VAS score compared to both of the other groups, while hyaluronic acid (HA) also showed a significantly lower VAS score compared to the control group. Both MEBO and HA resulted in a statistically significant reduction in total analgesic consumption. No significant differences were observed between the groups regarding wound size.
Koca-Unsal et al., 2021 [37]	Randomized clinical trial	To explore the role of titanium-prepared platelet-rich fibrin (T-PRF) in promoting vascularization at the donor site in patients undergoing free gingival graft (FGG) procedures.	10 Turkish patients: - Test group (*n* = 5)—T-PRF membrane; - Control group (*n* = 5)—gelatin sponge.	Tissue thickness—intra-oral probing. Vascularization—pulsatility index (PI) in Doppler ultrasonography (US). US was performed before graft harvesting and on the 2nd, 4th, 7th, and 14th days following the FGG procedure.	20–53	The T-PRF group exhibited increased vascularity, which may contribute to enhanced healing of the soft tissue.
Bitencourt et al., 2021 [38]	Randomized, placebo-controlled trial with a parallel design and triple blinding	To assess the impact of photobiomodulation therapy (PBMT) at the donor site on patient morbidity and wound healing after free gingival graft (FGG) harvesting.	44 Brazilian patients: - Test group (*n* = 22)—PBMT; - Control group (*n* = 22)—placebo.	Primary outcome—postoperative pain using VAS. Secondary outcomes: - “Analgesic consumption”; - “Patient-reported outcome measures (PROMs): general function, oral function, and other symptoms (swelling, bleeding etc.)”; - “Wound closure evaluation—dental cotton roll with two tone disclosing dye solution (Mira-2-Ton^R^)”.	≥21	Postoperative pain: No differences were observed in the VASLOG scores in the placebo group, whereas the PBMT group showed significant differences at 6, 24, 48, and 72 h. Analgesic consumption was notably higher in the placebo group. The PBMT group reported significantly better general and oral function. At 7 days, the PBMT group demonstrated significantly better palatal wound closure compared to the placebo group.
Miguel et al., 2021 [39]	Double-blind, randomized clinical trial with a parallel design	To investigate the clinical, immunological, and patient-centered effects of microcurrent electrotherapy on the healing of palatal wounds.	53 Brazilian patients: - Control (Sham) group (*n* = 27)—false application of electrotherapy; - Test (EE) group (*n* = 27)—application of electrotherapy.	Clinical measurement (90-day evaluation): - “Remaining wound area (RWA)”, “epithelialization (E)”, “scar and tissue colorimetry (STC)”—standardized photographs; - “Tissue thickness (TT)”—endodontic spreader; - Early wound healing index and tissue edema. Patient-centered parameters (90-day assessment): - “Oral Health Impact Profile (OHIP)”—Likert scale; - “Number of analgesics (NA)”; - “Self-reported pain (VAS)”; - Immunologic Analysis—inflammatory markers (pg/mL).	33–56	The EE group experienced faster wound closure and epithelialization at 7 and 14 days post-harvest compared to the Sham group. Fewer instances of pain were reported in the EE group than in the Sham group at the 3-day follow-up. Similarly, the EE group reported improvements in OHIP scores just 2 days after the procedure. Electrotherapy led to modulation of inflammatory markers associated with wound healing.
Spin et al., 2021 [40]	A pilot randomized controlled trial	To assess the effectiveness of a specially designed latex membrane as a physical barrier to promote wound healing, epithelialization, and reduce pain in the palatal region following free gingival graft (FGG) surgery.	24 Brazilian patients: - Control group (*n* = 14)—acrylic plate associated with surgical cement; - Test group (*n* = 10)—latex membrane group.	Wound healing area—using standardized digital photographs. Epithelization—3% oxygen peroxide test. Bleeding (present/absent). Self-reported pain (VAS).	30–70	By day 15, patients in the control group had fully healed wounds, while the latex group showed a mean wound closure of 98.6%. By day 30, both groups achieved complete wound closure. At day 7, 21.4% of patients in the control group experienced bleeding, while none of the patients in the latex group showed any bleeding. In terms of reported pain, VAS scores were higher in the control group, though the difference was not statistically significant.
Yussif et al., 2021 [41]	A single-center randomized controlled trial with two parallel study groups	To compare the effectiveness of propylene mesh as a protective covering with that of a conventional custom-made acrylic stent after palatal graft harvesting.	Two groups of 12 Egyptian patients with 24 sites (four sites were excluded from the study as dropouts): - Test group—propylene mesh; - Control group—custom-made acrylic palatal stent.	Trans-operative and postoperative outcomes—VAS scale (30 days): - “Bleeding amount”; - “Bleeding duration”; - “Pain intensity”. Postoperative outcomes (30 days): - “Pain duration” (VAS scale); - “Patient satisfaction” and complications assessment by operator (questionnaire); - “Healing period” (scale 1–30 days); - “Healing profile” (Healing index).	22–33	The polypropylene mesh proved more effective in reducing both bleeding and pain. The reduction in bleeding volume and its duration with the propylene mesh was statistically significant. No significant difference was found between the groups in terms of patient satisfaction or the duration of the healing process. The healing profile of the test group showed statistically significant improvement compared to the control group.
Miguel et al., 2021 [42]	A randomized, single-blind clinical study	To investigate the clinical, immune response, and patient-reported outcomes of applying enamel matrix protein derivative (EMD) to excisional wounds in the palatal mucosa.	44 patients: - EMD group (*n* = 22); - Control group (*n* = 22).	Clinical parameters: - “Remaining wound area (RWA)”—measurements obtained from standard photographs; - “Tissue thickness (TT)”—measurement using endodontic spreader. Patient-centered parameters: - “Oral Health Impact Profile scores” (questionnaire using Likert scale answers); - “Number of analgesics (NA) consumed”; - “Self-reported pain—VAS”. Immunologic parameters.	33–67	No significant differences were found between the groups in terms of RWA and re-epithelialization. Likewise, no significant inter-group differences were observed in NA and Oral Health Impact Profile scores. EMD seemed to specifically influence the levels of monocyte chemoattractant protein-1, macrophage inflammatory protein-1α, matrix metallopeptidase 9, and tissue inhibitor of metalloproteinases-2 in wound fluids.
Basma et al., 2022 [43]	Clinical trial with a parallel design	To evaluate patient-reported outcomes associated with four distinct wound dressings placed on the palatal donor site after harvesting a free epithelialized mucosal graft (FEG).	72 American patients: - Control group (*n* = 18)—collagen plug + sutures (CPS); - CPC group (*n* = 18)—collagen plug with cyano-acrylate; - PRF group (*n* = 18)—platelet rich fibrin + sutures; - PS group (*n* = 18)—palatal stent only.	Assessment of the following parameters over a 14-day period: Pain perception using VAS; Number of analgesics consumed; The amount of swelling; Tolerance to physical activity; Readiness for additional treatment.	33–76	All test groups, when compared to the control group, reported significantly lower pain perception, reduced analgesic consumption, and greater willingness for retreatment. No significant differences were observed among the test groups. The PS group recorded the lowest overall pain scores. Patient morbidity did not seem to be influenced by palatal thickness, graft length, graft width, or graft thickness.
Alasqah et al., 2022 [44]	Prospective randomized case–control study	To investigate the clinical advantages and effects of using honey as a dressing for promoting palatal wound healing after free gingival graft (FGG) harvesting.	20 Saudi patients: - Test group (*n* = 10)—Medihoney; - Control group (*n* = 10)—no dressing material.	Measurement of length and width of the donor site.	35 (mean age)	In the first week, a significant difference was observed in the proportion of patients showing donor site healing, with 56% in the test group compared to 44% in the control group. At 4 weeks, the test group showed a donor site healing percentage of 86% in width and 91% in length, while the control group had 14% healing in width and 9% in length.
Morshedzadeh et al., 2022 [45]	Randomized, controlled, triple-blind split-mouth clinical study	To evaluate the impact of photobiomodulation (PBM) on enhancing postsurgical wound healing and alleviating pain and discomfort at the palatal donor site, using a 940 nm GaAlAs laser (0.21 W, 5 J/cm^2^) in continuous wave mode.	16 Iranian patients requiring bilateral free gingival grafts (FGG): - Test group (*n* = 16) –LLLT GaAlAs, 940 nm, 5 J/cm^2^ treatment right after surgery; - Control group (*n* = 16)—received sham irradiation.	Remaining wound area (RWA)—standard photographs using Canon 70D. Epithelialization—drippling hydrogen peroxide (3%). Pain and discomfort—based on the VAS. Bleeding—presence or absence. Color match—measured by Adobe Photoshop CC2017.	29–65	The test group showed a significantly smaller RWA compared to the control group on days 7 and 14 post-surgery. Bleeding was more pronounced in the test group than in the control group on the day of the surgery. No significant differences in pain or discomfort were observed between the groups during the 11 days following surgery, nor were there any differences in color match scores on days 28 and 60 post-surgery.
Oliveira et al., 2023 [46]	Quasi-randomized controlled clinical study with a parallel design and blinded examiner	To investigate how a gel with green tea extract and hyaluronic acid (HA) influences pain relief and promotes the healing of palatal donor sites after the removal of a free gingival graft (FGG).	42 Brazilian patients: - Control group (*n* = 14)—suturing; - Placebo group (*n* = 14)—“vehicle gel” application; - Test group (*n* = 14)—“gel containing green tea extract and HA”.	Evaluation of the following parameters over a 4-week period: Clinical measurements (wound size). Complete wound epithelialization (CWE) using H_2_O_2_. Color match, by the visual analog scale (VAS). Postoperative pain using VAS.	18–60	All groups exhibited a similar gradual reduction in wound size. No significant differences were observed between the groups in terms of complete wound epithelialization or pain levels.
Alkaya et al., 2023 [47]	Single-center, double-blind, randomized controlled clinical trial (RCT) with two groups	o evaluate the impact of a pre-operatively crafted, zinc-infused surgical stent (ZN) versus suturing with a gelatin-based hemostatic agent (HA) on palatal wound healing and patient recovery after free gingival graft (FGG) procedures.	52 Turkish patients: - Control group (*n* = 28)—absorbable hemostatic gelatin sponge sutured with a single compression suture (HA group); - Test group (*n* = 24)—Zn-containing polymer as a stent for palatal region (ZN group).	The total duration of the surgery was recorded. Evaluation of the following parameters over a 56-day period: - PP (postoperative pain); BS (burning sensation) and DH (changes in dietary habits) using a VAS. - DB (delayed bleeding). - Completion of re-epithelialization (CE) was clinically evaluated by H_2_O_2_.	≥19	Overall surgical time and donor surgical time were shorter in ZN group. The ZN group showed significantly lower postoperative pain (PP), delayed bleeding (DB), burning sensation (BS), and changes in dietary habits (DH), along with faster re-epithelialization.
Loureiro et al., 2023 [48]	Prospective, longitudinal, triple-blind, randomized clinical trial with a parallel design and placebo control	To clinically and immunologically assess the impact of ozonated oil on the healing of palatal wounds.	28 Brazilian patients: - Control group (*n* = 14)—“non-ozonated sunflower oil (placebo)”; - Test group (*n* = 14)—“ozonized seed sunflower oil with peroxide index between 510 and 625 meq”.	The primary outcome was the size of the remaining palatal scar (mm^2^). Immunological assessment was considered a secondary outcome.	18–60	No significant differences were observed in the wound area measurements (mm^2^) between the groups across the various time periods. In relation to TGF-ß and 4-HNE there were no differences between groups and periods. There was no significant difference in VEGF levels between the groups, but a significant change was observed within the test group between days 3 and 7, showing a reduction in VEGF on day 7.
Alpan and Cin, 2023 [49]	Prospective, randomized controlled clinical trial with parallel group design	to assess the impact of topical hyaluronic acid (HA), hypochlorous acid (HOCl), and flurbiprofen on postoperative complications and recovery at palatal donor sites following free gingival graft (FGG) surgery.	60 Turkish patients: - “Control group” (*n* = 15); - “HA group” (*n* = 15); - “HOCl group” (*n* = 15); - “Flurbiprofen group” (*n* = 15).	The primary outcome—the palatal wound healing using the “Landry Wound Healing Index (WHI)” to 28th day postoperatively. The secondary outcome—“perception of pain”, “discomfort while chewing”, and “burning sensation” using VAS. Delayed bleeding (+/−). Complete epithelialization (CE)—using H_2_O_2_.	18–48	The HA group showed better wound healing index (WHI) on days 7, 14, and 21 compared to the other groups. Complete epithelialization (CE) occurred on day 21 in the HA group, while it was observed on day 28 in the other groups. No significant differences were found in WHI between the HOCl, flurbiprofen, and control groups. The HOCl group reported the lowest visual analog scale (VAS) pain scores across all time points. No delayed bleeding (DB) was observed in any of the groups. The groups treated with topical agents required significantly fewer analgesics than the control group.
Gatti et al., 2023 [50]	Double-center, parallel arm, randomized controlled clinical trial	To evaluate postoperative patient discomfort and surgical complications using leucocyte- and platelet-rich fibrin (L-PRF) membranes or hemostatic agents applied at palatal wound after FGG collecting.	42 patients: - Test group—L-PRF membrane (*n* = 21); - Control group—hemostatic agent (*n* = 21).	Primary outcome—postoperative pain associated with the surgical wound at the palatal site. Secondary outcome: - “Postoperative pain” based on analgesics consumption—VAS; - “Postoperative discomfort”—VAS; - “Inability to chew”—VAS; - “Postoperative stress”—VAS; - “Surgical chair time”; - “The thickness of palatal fibromucosa and FGG”.	29–43	One week after surgery, a statistically significant difference was observed between the groups regarding postoperative stress. No significant differences were found between the groups in terms of postoperative pain, patient discomfort, chewing difficulty, or surgical chair time. There were no significant differences between the groups regarding the thickness of the palatal fibromucosa or the free gingival graft (FGG).
Juber et al., 2023 [51]	Randomized controlled clinical trial	To evaluate how highly ozonated sunflower oil influences postoperative pain and overall quality of life in patients undergoing free gingival graft (FGG) harvesting from the palatal donor site.	28 Brazilian patients: - Control group (*n*-14)—non-ozonated sunflower oil; - Test group (*n* = 14)—highly ozonated sunflower oil.	All participants completed questionnaires at day 7, including the “Oral Health Impact Profile (OHIP-14)” to assess quality of life and the “Visual Analog Scale (VAS)” to evaluate postoperative pain.	40–65	No statistically significant differences in postoperative pain were observed between the groups at any time point. A significant positive correlation was found between pain levels and analgesic consumption in both groups. Participants in the test group reported higher overall scores on the OHIP-14 compared to the control group, particularly regarding diet dissatisfaction, meal interruptions, and irritability. MDA levels measured 7 days post-treatment were higher in the test group than in the control group.
Tabari et al., 2023 [52]	A randomized, double-blind split-mouth clinical study	To investigate the effects of a propolis-based oral ointment on pain relief and wound healing at the palatal donor site after free gingival graft (FGG) harvesting.	10 Iranian patients (20 sites): - Control group (*n* = 10)—placebo; - Test group (*n* = 10)—5% propolis oral ointment.	Patients were re-evaluated on days 1, 3, 7, 14, and 21 post-surgery to assess burning sensations and pain at the donor site. The color match between the donor site and surrounding healthy tissue was assessed on days 7, 14, 21, 30, and 42. Bleeding at the donor site was examined on day 7. Tissue thickness at the donor site was measured on days 0, 30, and 42.	28–54	Pain scores on days 1 and 3 were significantly lower in the test group. No significant difference in burning sensation was observed between the two groups. The color match in the test group was significantly better on days 14, 21, 30, and 42. No significant difference was found in terms of donor site bleeding or tissue thickness between the groups.
Scott et al., 2024 [53]	A randomized controlled study with a two-arm, parallel design	To compare the healing of the palatal tissue donor site when platelet-rich fibrin (PRF) is applied as a wound dressing versus the use of a hemostatic agent.	74 American patients: - Control group (*n* = 37)—hemostatic agent group; - Test group (*n* = 37)—PRF group.	Patient pain assessment and analgesic consumption were documented using a 21-point numerical scale (NMRS-21) at 24, 48, and 72 h post-surgery. Palatal early healing index (PEHI) scores, including wound color, epithelialization, presence or absence of swelling, granulation tissue, and bleeding on gentle palpation, were generated.	56 (mean age)	NMRS-21 pain scores indicated a notable decrease in pain over time in both groups, with no significant difference observed between the groups at any point. No significant differences were found between the groups regarding analgesic consumption at 24, 48, and 72 h. Both groups showed significant improvement in PEHI scores over the 4-week period, but there was no significant difference between the groups at any time point (1, 2, 3, 4 weeks).
Yadav et al., 2024 [54]	A randomized clinical trial	To assess the impact of a chitosan-based dressing (CD) on promoting early wound healing and controlling hemostasis at palatal donor sites following FGG procedure.	28 Indian patients: - Control group (*n* = 14)—2 min compression using wet gauze (WG); - Test group (*n* = 14)—WG + CD.	“Complete epithelialization (CE)” and “color match (CM)” were defined. Right after graft harvesting, “immediate bleeding (IB)” was noted (yes/no). The patients recorded “delayed bleeding (DB)” (for 1 week), “number of analgesic tablets taken”, and “VAS pain scores” (for 2 weeks) on a daily basis.	18–65	The test group (TG) exhibited higher prevalence of complete epithelialization (CE) at weeks 2 and 3, as well as higher VAS scores for color match (CM). However, the statistically significant difference between groups was only observed for CM at week 4. Fewer patients in the TG experienced inflammatory bleeding (IB) and delayed bleeding (DB). Although the TG reported higher average pain scores and analgesic consumption up to day 5, no statistically significant differences were found between the two groups at any time point.

**Table 2:** A summary of research studies using platelet-rich fibrin (PRF) for palate protection.

Author, Year of Publication	Number of Subjects	Control Group	Does PRF Offer Benefits Compared to the Control Group Concerning Specific Parameters?						
			Color Match (CM)	Wound Epithelialization (Healing)	Post-Op Bleeding	Pain Perception (VAS Scale)	Post-Op Discomfort	Feeding Habbits (FH)	Analgesics Cosumption
Ustaoglu et al., 2016 [21]	34 Turkish patients	Untreated	Yes	Yes	Yes	No	No data	No data	No
Femminella et al., 2016 [22]	40 Italian patients	Absorbable gelatin sponge	No data	Yes	No	No data	Yes	Yes	No
Ozcan et al., 2017 [26]	125 Turkish patients	2 groups: Butyl-cyano-acrylate and gauze compression	No data	Yes	Yes	Yes	No data	Yes	No data
M. A. Baham-mam, 2018 [30]	24 Saudi patients	Non-eugenol periodontal pack with sutures	Yes	No data	No data	Yes	Yes	No data	No data
Patara-pongsanti et al., 2019 [31]	18 Thai patients	Oxidized regenerated cellulose (ORC)	No data	Yes	No data	Yes	Yes	No data	No data
Sharma et al., 2020 [32]	20 Indian patients	Collagen membrane	No data	No	No	No	No data	No data	No data
Koca-Unsal et al., 2021 [37]	10 Turkish patients	Absorbable gelatin sponge	No data	Yes	No data	No data	No data	No data	No data
Basma et al., 2022 [43]	72 American patients	Collagen plug	No data	No data	No	Yes	No data	No data	Yes
Gatti et al., 2023 [50]	42 patients	Oxidized regenerated cellulose (ORC)	No data	No data	No data	No	No	No data	No
Scott et al., 2024 [53]	74 American patients	Oxidized regenerated cellulose (ORC)	No data	No	No data	No	No data	No data	No

**Table 3:** An overview of research on the use of photobiomodulation following graft harvesting.

Author, Year of Publication	Number of Subjects	Control Group	Does Photobiomodulation Offer Benefits Compared to the Control Group Concerning Specific Parameters?						
			Color Match (CM)	Wound Epithelialization (Healing)	Post-Op Bleeding	Pain Perception (VAS Scale)	Post-Op Discomfort	Feeding Habbits (FH)	Analgesics Cosumption
Ustaoglu et al., 2017 [23]	35 Turkish	Sham group	Yes	Yes	Yes	No	No data	No data	No
Isler et al., 2018 [28]	36 Turkish patients	Spontaneous healing	No data	Yes	No data	No	Yes	No	No
Bitencourt et al., 2021 [38]	44 Brazilian patients	Placebo	No data	Yes	No data	No	Yes	Yes	Yes
Morshe-dzadeh et al., 2022 [45]	16 Iranian patients	Sham irradiation	No	Yes	No	No	No	No data	No data

**Table 4:** Classification of palate wound management techniques after graft harvesting, according to their source origin.

Biological		Physical	Chemical
Animal Origin	Plant Origin		
Platelet-rich fibrin (PRF) [21,22,26,30,31,32,37,43,50,53] Erythropoietin (EPO) [27] Enamel matrix protein derivative (EMD) [42] Medihoney (propolis) [44,52] Chitosan gel [24,54]	Oxidized regenerated cellulose [17,31] Medical plant extract (MPE) [19] Moist exposed burn ointment (MEBO) [36] Green tea extract [46]	Photobiomodulation (PBM) [20,23,28,38,45] Non-thermal atmospheric pressure plasma (NAPP) [33] Microcurrent electrotherapy [39]	Ozonated oil [18,48,51] Simvastatin [24] Flurbiprofen [29,49] Alvogyl [34] Phenytoin (PHT) [35] Hyaluronic acid (HA) [25,46,49] Latex membrane [40] Propylene mesh [41] Cyanoacrylate [26,43] Zinc-containing surgical stent (ZN) [47] Hypochlorous acid (HOCl) [49]

## Data Availability

No new data were created or analyzed in this study. Data sharing is not applicable to this article.

## Supplementary Materials

The following supporting information can be downloaded at: https://www.mdpi.com/article/10.3390/jfb15120360/s1, Supplementary file: PRISMA 2020 manuscript checklist.
